# Tradeoff between Biomass and Flavonoid Accumulation in White Clover Reflects Contrasting Plant Strategies

**DOI:** 10.1371/journal.pone.0018949

**Published:** 2011-04-19

**Authors:** Rainer W. Hofmann, M. Z. Zulfiqhar Jahufer

**Affiliations:** 1 Lincoln University, Faculty of Agriculture and Life Sciences, Lincoln, New Zealand; 2 AgResearch, Grasslands Research Centre, Palmerston North, New Zealand; Purdue University, United States of America

## Abstract

An outdoor study was conducted to examine relationships between plant productivity and stress-protective phenolic plant metabolites. Twenty-two populations of the pasture legume white clover were grown for 4½ months during spring and summer in Palmerston North, New Zealand. The major phenolic compounds identified and quantified by HPLC analysis were glycosides of the flavonoids quercetin and kaempferol. Multivariate analysis revealed a trade-off between flavonoid accumulation and plant productivity attributes. White clover populations with high biomass production, large leaves and thick tap roots showed low levels of quercetin glycoside accumulation and low quercetin:kaempferol ratios, while the opposite was true for less productive populations. The latter included stress-resistant ecotypes from Turkey and China, and the analysis also identified highly significant positive relationships of quercetin glycoside accumulation with plant morphology (root:shoot ratio). Importantly, a high degree of genetic variation was detected for most of the measured traits. These findings suggest merit for considering flavonoids such as quercetin as potential selection criteria in the genetic improvement of white clover and other crops.

## Introduction

The legume white clover (*Trifolium repens* L.) is a key nitrogen-fixing species in many pasture systems of the world [1]. White clover growth and yield is markedly reduced during summer, when the species is exposed to a wide spectrum of interacting stress factors [2], [3]. These include annual peak levels of ultraviolet (UV) radiation, periods of limited availability of water and nutrients, high temperatures, increased pressure from grazing, herbivory and diseases, as well as from other abiotic and biotic factors. This situation is likely to be exacerbated in the context of global climate change. Flavonoids have been implicated with plant resistance to many of these stress factors. This can be attributed to the wide array of functions for these secondary plant metabolites, including UV-screening, energy dissipation, antioxidant and herbivore-deterring capacities [4]–[7]. Plants exposed to stress frequently display morphological changes that help minimize damaging effects, both above- and belowground. This can include reductions in leaf size and in biomass accumulation, as well as increases in the root:shoot ratio under a variety of limiting factors [8]–[11].

Ecological plant strategy theory [12] implies that species investing into biochemical means of stress protection (“stress tolerators”) are likely to invest less carbon into constitutive productivity. “Competitors” are plant species showing the opposite strategy, with investment into the accumulation of biomass rather than of biochemical compounds for stress protection. Examinations of these relationships can be compounded by species-specific differences and this can be addressed by intraspecific studies conducted at the population level. However, there is a lack of large-scale population studies that examine such relationships, particularly the possible involvement of flavonoids. We hypothesised that flavonoid accumulation would come at a cost for productivity attributes in white clover populations grown under New Zealand outdoor conditions. For this purpose, 22 white clover populations diverse in morphology were grown during spring and summer, and subsequently characterized for selected morphological characteristics, as well as foliar flavonoid accumulation. A further objective was to assess the magnitude of genotypic variation for the traits examined among the 22 white clover populations. This would provide information on potential genetic variation that could be used for future cultivar development.

## Materials and Methods

A total of 22 white clover populations were evaluated in the study (Table 1). The 22 populations included 18 commercial cultivars, two breeding lines and two ecotypes (Sarikamis and Tienshan). The experimental layout was a randomised complete block design with two replicates. Each population was represented by a random sample of 12 genotypes per replicate.

**Table 1:** Description of leaf sizes for the 22 white clover populations evaluated in the experiment.

Leaf size					
Very Large	Large	Medium-large	Medium	Small-medium	Small
Tillman II	Aran	Sustain	Huia	Prestige	Tienshan
	Will	Tribute	Crusader	Patriot	Nomad
	Kopu II	Emerald	Bounty	Durana	
	Barblanca	Quest	Saracen	Sarikamis	
		Trophy	Breeding Line 1		
			Breeding Line 2		

**Figure 1:**
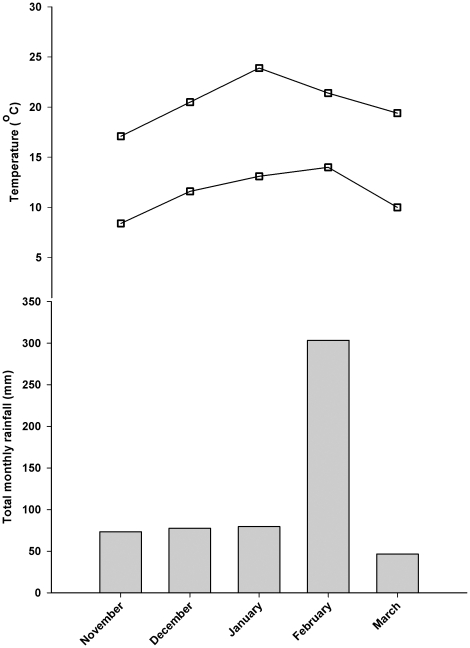
Mean monthly maximum and minimum temperatures (°C) and total monthly rainfall (mm) during the trial period from November 2003 to March 2004, at Palmerston North in New Zealand.

Plants were established in seedling mix in the glasshouse for two weeks and then transplanted for the outdoor study into cylindrical clay tiles that were 500 mm long and 150 mm wide to facilitate vertical growth of the white clover roots (one seedling per tile). The soil medium in the tiles was Egmont black loam from Taranaki, New Zealand [13]. Plants were grown outdoors from mid-spring and during summer (12 November 2003–21 March 2004), at the AgResearch Grasslands Research Centre in Palmerston North, New Zealand (40° 21′ S, 175° 37′ E). A local meteorological station recorded climatic data, and the mean monthly maximum and minimum temperatures, as well as total monthly rainfall for the trial period are presented in Figure 1. Additional moisture was supplied to the plants with a sprinkler system to prevent wilting during low rainfall periods. The plants were actively growing throughout the experimental period and were defoliated to a height of 30 mm using hand clippers every 3–4 weeks to mimic grazing and to prevent plants from outgrowing the tiles.

### Measurements

At the end of the experimental period (3rd week of March, three weeks after the final defoliation), plants were harvested for morphological and biochemical analysis. For the latter, fully unfolded white clover leaf laminae were collected, ground in liquid nitrogen and 50 mg freeze-dried powder extracted in 3 mL acidified methanol (MeOH:H_2_O:HOAc at 89∶10∶1), as described previously [14]. The extracts were examined in high performance liquid chromatography (HPLC) with photodiode array (PDA) monitoring for identification and quantification of flavonoids. The HPLC gradient consisted of solvent A [1.5% H_3_PO_4_] and solvent B [HOAc:CH_3_CN:H_3_PO_4_:H_2_O (20∶24∶1.5∶54.5)], mixed using a linear gradient starting with 80% A, decreasing to 33% A at 30 min, 10% A at 33 min and 0% at 39.3 min. HPLC peak identification and calculations followed established methods [14].

At final harvest, digital calipers were used to measure the taproot diameter at the base of each plant. Calipers were also used to determine the average width of the central leaflet of three first fully unfolded leaves, chosen from three randomly selected stolons per plant. Leaflet diameter provides an accurate reflection of leaf size in white clover [15]. Total aboveground shoot material per plant was weighed after drying at 80°C for 48 h. Roots were washed from the soil and weighed after drying at 80°C for 48 h to establish the root:shoot ratio for each individual plant.

### Data analysis

The experimental data were analyzed with the objective of estimating the magnitude and type of genotypic variation among the 22 white clover populations for the eight traits measured. The analysis was conducted using the variance component analysis procedure, Residual Maximum Likelihood (REML) option, in Genstat [16]. A completely random linear model was used in the analyses using the REML algorithm. The final genotypic means were based on Best Linear Unbiased Predictors (BLUPs) [17]. The genotypic variance components estimated for the traits using REML were used to estimate repeatability (*R*) [18] on a line (population) mean basis. Following the variance component analysis, the population-by-trait BLUP adjusted mean matrix was analyzed using a combination of cluster analysis and principal component analysis [19]–[21]. This analysis provided a graphical summary, on a multivariate basis, of the information on variation among the 22 populations relative to the eight traits under consideration in this study. Phenotypic correlation coefficients among the traits were calculated using Genstat [16].

## Results

**Figure 2:**
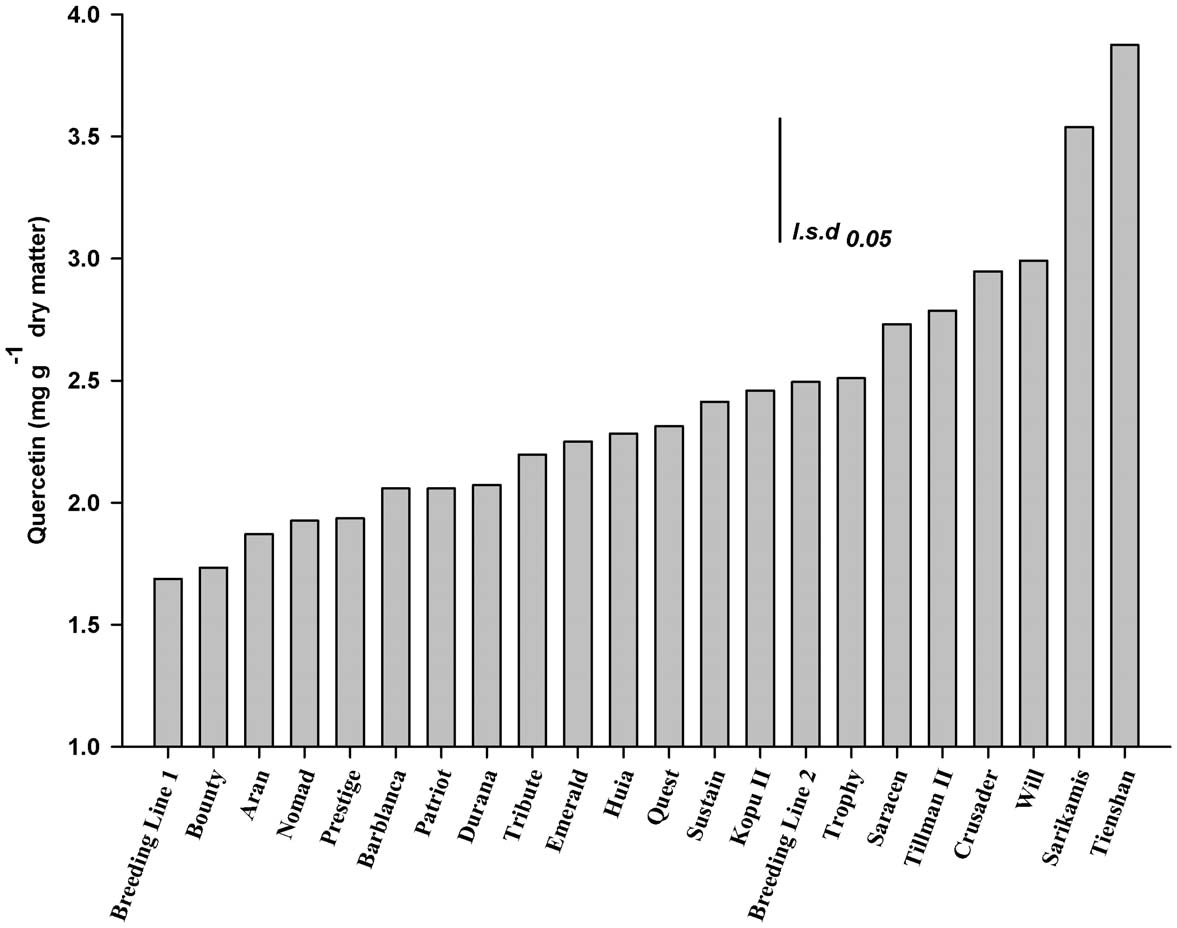
Quercetin glycoside accumulation in the leaves of 22 white clover populations.

The major flavonoids detected were glycosides of the flavonols kaempferol and quercetin. There was significant (P<0.05) genotypic variation among the 22 white clover populations for all the traits measured (Table 2). Line mean repeatability for flavonoid glycoside accumulation ranged from 15% for kaempferol to 46% for quercetin. The highest repeatability level was found for leaf width at 56% (Table 2). Figure 2 illustrates the range of variation among the 22 white clover populations for the trait quercetin. Compared to Breeding Line 1 and cultivar Bounty, quercetin glycoside levels were up to 90% higher in the cultivars Crusader and Will, and almost 150% higher in the ecotypes Tienshan and Sarikamis (Figure 2).

**Table 2:** Means, ranges, variance components (σ^2^_g_) with associated standard errors (±SE), and line mean repeatability (*R*) for traits measured in 22 white clover populations.

	Q (mg g^−1^)	K (mg g^−1^)	F (mg g^−1^)	Q:K ratio	Shoot weight (g)	Leaf width (mm)	Tap root diameter (mm)	Root:shoot ratio
Mean	2.42	1.17	3.59	2.61	2.82	17.3	4.45	0.37
Range	1.69–3.88	0.93–1.62	2.60–5.01	1.7–4.1	1.1–4.2	12.9–22.3	3.4–5.7	0.29–0.47
*l.s.d._0.05_*	*0.6*	*0.6*	*0.8*	*0.8*	*0.8*	*1.8*	*0.6*	*0.04*
σ^2^_g_	0.34±0.12	0.036±0.017	0.396±0.114	0.46±0.17	0.69±0.24	0.05±0.02	0.34±0.12	0.002±0.001
σ^2^_ε_	0.79±0.05	0.419±0.027	1.654±0.107	2.329±0.151	2.16±0.14	0.08±0.01	1.05±0.05	0.007±0.001
R	0.46	0.15	0.32	0.28	0.39	0.56	0.39	0.37

Q, quercetin; K, kaempferol; F, flavonols.

**Figure 3:**
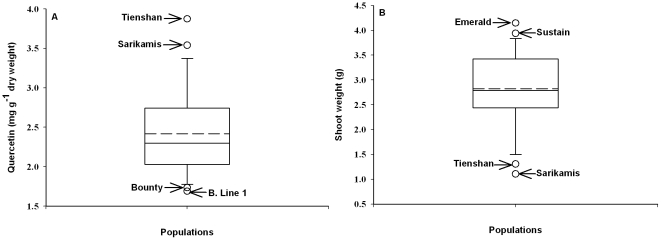
Box plots showing variation among the 22 white clover populations. (**A**) quercetin accumulation and (**B**) shoot weight. The top and bottom two populations for trait expression are indicated. B. line 1, Breeding Line 1. Mean (----).

**Figure 4:**
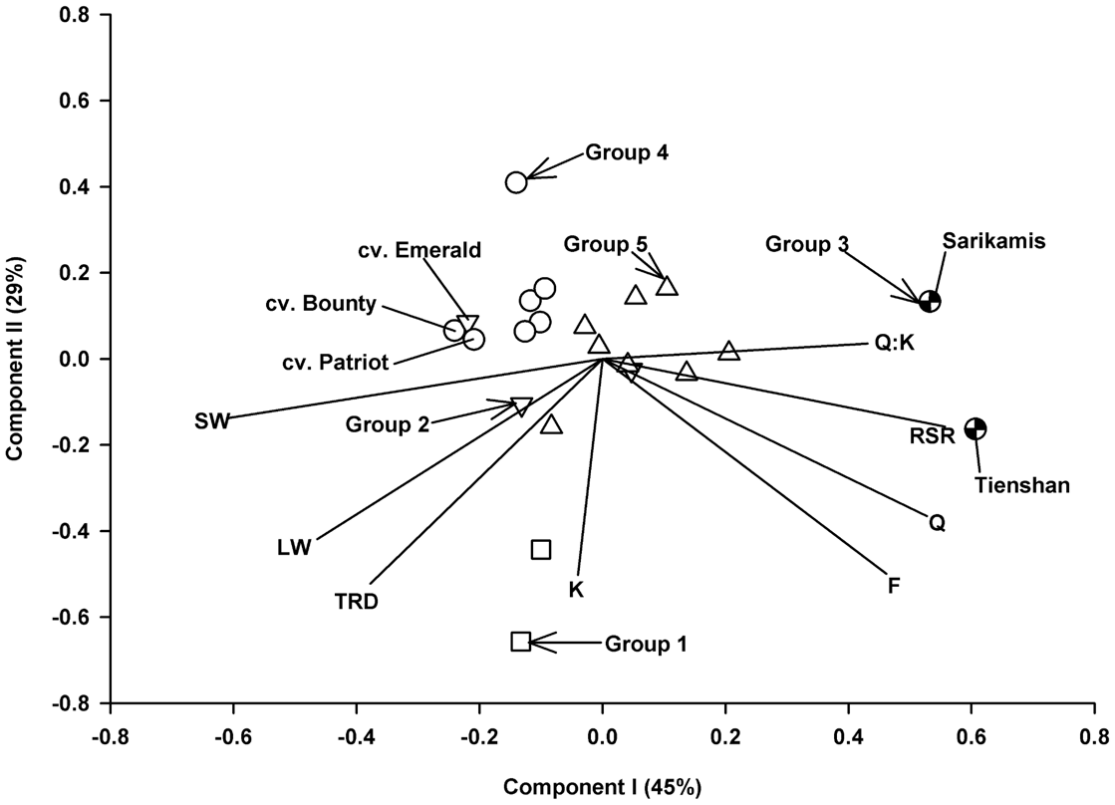
Biplot displaying component vectors I and II based on standardized BLUP values for eight morphological and biochemical traits, measured in 22 white clover populations. The symbols represent the five population groups generated from cluster analysis. The directional vectors represent the traits F, flavonols; K, kaempferol; LW, leaf width; Q, quercetin; Q:K, quercetin:kaempferol ratio; RSR, root:shoot ratio; SW, shoot weight; TRD, tap root diameter.

Comparison of quercetin glycoside accumulation and shoot weight among the 22 populations showed an inverse relationship between the two traits: the two ecotypes Tienshan and Sarikamis were highest in the accumulation of quercetin glycosides (Figure 3a) and lowest in shoot weight (Figure 3b). The principal components analysis (PCA) biplot (Figure 4) provided a graphical representation of the relationships between two sets of information (populations and traits), simultaneously. The correlation structure of the traits is indicated by the directional vectors in the biplot. The origin (0,0) represents the average point for all eight traits. In the biplot, the first and second principal components explained nearly 75% of the variance in the dataset, 45% from PC1 and 29% from PC 2 (Figure 4). Pairs of traits such as quercetin and root:shoot ratio, and shoot weight and leaf width, showed a strong positive association (directional vectors at <45°). In comparison, the pairs of traits quercetin and shoot weight, and root:shoot ratio and shoot weight showed strong negative associations (directional vectors approaching 180°). Estimated phenotypic correlation coefficients among the traits further supported the association among the vectors indicated in the biplot (Figure 4). For example, at a significance of P<0.05, the estimated phenotypic correlations between Q/F, Q/Q:K, Q/RSR, Q/SW, SW/LW and RSR/SW were 0.94, 0.69, 0.55, −0.45, 0.69 and −0.78, respectively.

The five population groups generated from cluster analysis of the 22 population ×8 trait mean matrix showed distinct separation with little overlap (Figure 4). Group 3 consisted of two ecotypes, Sarikamis and Tienshan, with high expression of the traits quercetin, flavonols, Q:K ratio and root:shoot ratio (Figure 4). Cultivars Bounty and Patriot from Group 4 and Emerald from Group 2 showed high expression of shoot weight (Figure 4). While Groups 1 and 3 had two members each, Group 5 had eight cultivars (Table 3). The two members of Group 3 can be characterized as populations having a combination of high quercetin and flavonol glycoside accumulation together with small leaves and low shoot production (Table 3). In comparison, Group 4 on average had low quercetin glycoside accumulation but more than 2.5 times the mean shoot weight of Group 3 (Table 3). The three members of Group 2 had intermediate quercetin accumulation, but were above average in expression of the traits shoot weight, leaf width and tap root diameter (Table 3). Group 1 was characterized by high average values for these morphological traits, as well as for kaempferol. Group 5 showed intermediate expression of most traits (Table 3).

**Table 3:** Within-group population means for each trait based on the five clusters generated from cluster analysis of the 22 white clover population × eight trait BLUP adjusted mean matrix.

Group	Group members	Q (mg g^−1^)	K (mg g^−1^)	F (mg g^−1^)	Q:K ratio	Shoot weight (g)	Leaf width (mm)	Tap root diameter (mm)	Root:shoot ratio
1	Tillman II	2.89	1.51	4.46	2.04	3.44	21.6	5.47	0.37
	Will								
2	Crusader	2.54	1.03	3.52	3.28	3.83	18.3	4.80	0.33
	Emerald								
	Sustain								
3	Sarikamis	3.71	1.11	4.72	3.79	1.21	13.5	3.71	0.44
	Tienshan								
4	Barblanca	1.96	1.11	3.09	2.29	3.12	17.3	4.37	0.33
	Bounty								
	Breeding Line 1								
	Durana								
	Patriot								
	Prestige								
	Tribute								
5	Aran	2.32	1.20	3.54	2.49	2.44	16.8	4.31	0.39
	Breeding Line 2								
	Huia								
	Kopu II								
	Nomad								
	Quest								
	Saracen								
	Trophy								

Q, quercetin; K, kaempferol; F, flavonols.

## Discussion

To our knowledge, this is the first study examining relationships between plant productivity and flavonoid accumulation under outdoor conditions and across a large number of populations within a species. The study was conducted under New Zealand summer conditions, and multivariate analysis of 22 white clover populations indicates a tradeoff between biomass and quercetin accumulation. This is in line with ecological plant strategy theory, suggesting that investment into productivity comes at a cost for the accumulation of compounds for biochemical stress protection [12].

The bi-variate association between quercetin glycoside accumulation and shoot weight (Figure 3a & 3b) was further elucidated when correlation among all the traits was examined on a multivariate basis using pattern analysis (Figure 4). One end of PC1 associated with plant attributes that contribute towards effective resource capture (large leaf size, taproot diameter and aboveground yield), whereas the opposite end of PC1 was linked to traits of relevance for stress resistance, including high root:shoot and Q:K ratios, and accumulation of quercetin (but not kaempferol) glycosides (Figure 4). Accumulation of kaempferol was not significantly related to that of quercetin glycosides and in contrast to quercetin, kaempferol also did not show associations to most other traits (Figure 4). Kaempferol is the monohydroxylated precursor of the *ortho*-dihydroxylated quercetin. A number of studies have indicated superior stress protection of B-ring-dihydroxylated flavonoids over their monohydroxylated counterparts, e.g. under high light [22], low temperatures [23] and elevated UV-B radiation [14]. Compared to their monohydroxylated counterparts, the extra hydroxyl group in *ortho*-dihydroxylated flavonoids may confer extra antioxidant capacity and can increase the dissipation of harmful energy [4], [24]. Quercetin has also been implicated in stress signaling and in the modulation of auxin transport [4], [25]. A higher root:shoot ratio represents increased carbon allocation into belowground – rather than aboveground – biomass that can contribute to plant resistance against a variety of limiting factors, e.g. under elevated UV-B radiation [9] or by increasing plant water status under drought [11].

Cluster analysis identified five distinctive population groups separated by differential morphology and flavonoid accumulation. Group 3, high in quercetin glycoside accumulation but low in yield, consisted of stress-resistant ecotypes from high altitude environments in China and Turkey. These ecotypes are exposed to a number of limiting factors in their natural habitats, including high UV radiation, cold temperatures and precipitation levels near the limit of white clover survival [9]. None of the white clover cultivars reached the quercetin glycoside levels in the two ecotypes in Group 3 (Figure 4). However, relatively high quercetin glycoside accumulation in some populations (Breeding Line 2, Trophy, Saracen, Tillman II, Crusader and Will) (Figure 2), may be a reflection of these populations being bred for dry environments [26], [27].

The range of data observed here indicates broad phenotypic variation among the populations for each trait (Table 1). The calculated line mean repeatability of the traits enabled estimation of upper limits of their degrees of genetic determination [18]. The high level of repeatability for leaf width is a reflection of the underlying genetic variation for leaf types among the 22 white clover populations evaluated in this study. The significant genotypic variation among the white clover populations for the flavonoid glycoside quercetin (Table 1) indicates potential for using this compound in breeding programs targeted at improving stress resistance.

To conclude, this study used flavonoids as the biochemical model to examine principles of plant strategy theory. The findings support our hypothesis of a trade-off between the production of plant biomass and of secondary metabolites of relevance for stress resistance: populations bred for – or originating from – stressed habitats typically contained high levels of specific protective flavonoids (quercetin glycosides) but tended to show low levels of constitutive productivity. For the purposes of this study, the white clover populations were all grown across the same environment. The results suggest merit for future genotype × environment stress studies in white clover and other crop species to investigate the potential role of quercetin as a key metabolic marker, e.g. for drought resistance. The genetic variation detected here will be valuable for the development of stress-resistant forage cultivars, e.g. via selection or breeding of populations that are productive and contain high levels of key protective metabolites. Accordingly, selected genotypes from the two high quercetin-expressing ecotype populations, Sarikamis and Tienshan, are currently being crossed with white clover lines of agronomic importance. The underlying genetic mechanisms affecting the negative association between biomass and quercetin in white clover are unclear. This could be due to a pleiotropic effect, closely linked genes or the genes for biomass and quercetin accumulation being linked in repulsion phase. Work on defining the genetics of this relationship and also the identification of quantitative trait loci for the two traits in white clover is currently underway.

## References

[1] Andrews M, Scholefield D, Abberton MT, McKenzie BA, Hodge S (2007). Use of white clover as an alternative to nitrogen fertiliser for dairy pastures in nitrate vulnerable zones in the UK: productivity, environmental impact and economic considerations.. Annals of Applied Biology.

[2] Hofmann RW, Campbell BD, Fountain DW (2003). Sensitivity of white clover to UV-B radiation depends on water availability, plant productivity and duration of stress.. Global Change Biology.

[3] Wery J (2005). Differential effects of soil water deficit on the basic plant functions and their significance to analyse crop responses to water deficit in indeterminate plants.. Australian Journal of Agricultural Research.

[4] Agati G, Tattini M (2010). Multiple functional roles of flavonoids in photoprotection.. New Phytologist.

[5] Buer CS, Imin N, Djordjevic MA (2010). Flavonoids: new roles for old molecules.. Journal of Integrative Plant Biology.

[6] Carlsen SCK, Fomsgaard IS (2008). Biologically active secondary metabolites in white clover (*Trifolium repens* L.) - a review focusing on contents in the plant, plant-pest interactions and transformation.. Chemoecology.

[7] Hofmann RW, Swinny EE, Bloor SJ, Markham KR, Ryan KG (2000). Responses of nine *Trifolium repens* L. populations to ultraviolet-B radiation: differential flavonol glycoside accumulation and biomass production.. Annals of Botany.

[8] Hofmann RW, Campbell BD (2011). Response of *Trifolium repens* to UV-B radiation: morphological links to plant productivity and water availability.. Plant Biology in press.

[9] Hofmann RW, Campbell BD, Fountain DW, Jordan BR, Greer DH (2001). Multivariate analysis of intraspecific responses to UV-B radiation in white clover (*Trifolium repens* L.).. Plant, Cell and Environment.

[10] Maggio A, Raimondi G, Martino A, De Pascale S (2007). Salt stress response in tomato beyond the salinity tolerance threshold.. Environmental and Experimental Botany.

[11] Wittenmyer L, Merbach W (2005). Plant responses to drought and phosphorus deficiency: contribution of phytohormones in root-related processes.. Journal of Plant Nutrition and Soil Science.

[12] Grime JP (2001). Plant Strategies, Vegetation Processes, and Ecosystem Properties..

[13] NZ Soil Bureau (1968).

[14] Hofmann RW, Campbell BD, Bloor SJ, Swinny EE, Markham KR (2003). Responses to UV-B radiation in *Trifolium repens* L. - physiological links to plant productivity and water availability.. Plant, Cell and Environment.

[15] Caradus JR, Chapman DF (1996). Selection for and heritability of stolon characteristics in two cultivars of white clover.. Crop Science.

[16] Genstat (2003). Genstat for Windows, Release 7.1 Reference Manual;.

[17] White TL, Hodge GR (1989). Predicting breeding values with applications in forest tree improvement..

[18] Falconer DS, Mackay TFC (1996). Introduction to quantitative genetics..

[19] Gabriel KR (1971). The biplot graphical display of matrices with application to principal component analysis.. Biometrika.

[20] Kroonenberg PM (1994). The TUCKALS line - a suite of programs for 3-way data analysis.. Computational Statistics & Data Analysis.

[21] Watson SL, DeLacy IH, Podlich DW, Basford KE (1995). GEBEIL: An analysis package using agglomerative hierarchical classificatory and SVD ordination procedures for genotype × environment data. Centre for Statistics Research Report..

[22] Agati G, Stefano G, Biricolti S, Tattini M (2009). Mesophyll distribution of ‘antioxidant’ flavonoid glycosides in *Ligustrum vulgare* leaves under contrasting sunlight irradiance.. Annals of Botany.

[23] Albert A, Sareedenchai V, Heller W, Seidlitz HK, Zidorn C (2009). Temperature is the key to altitudinal variation of phenolics in *Arnica montana* L. cv. ARBO.. Oecologia.

[24] Smith GJ, Markham KR (1998). Tautomerism of flavonol glucosides - relevance to plant UV protection and flower colour.. Journal of Photochemistry and Photobiology A - Chemistry.

[25] Peer WA, Murphy AS (2007). Flavonoids and auxin transport: modulators or regulators?. Trends in Plant Science.

[26] Jahufer MZZ, Clements R, Durant R, Woodfield DR (2009). Evaluation of white clover (*Trifolium repens* L.) commercial cultivars and experimental synthetics in south-west Victoria, Australia.. New Zealand Journal of Agricultural Research.

[27] Woodfield DR, Clifford PTP, Cousins GR, Ford JL, Baird IJ (2001). Grasslands Kopu II and Crusader: new generation white clovers.. Proceedings of the New Zealand Grassland Association.

